# Ligand-receptor pair-based signature score derived from on-treatment tumor specimens predicts immune checkpoint blockade response in metastatic melanoma

**DOI:** 10.1007/s12672-026-04396-4

**Published:** 2026-01-09

**Authors:** Huancheng Zeng, Rendong Zhang, Qiongzhi Jiang, Jundong Wu, Zhemin Zhuang, Yutong Fang

**Affiliations:** 1 Department of Breast Surgery, Cancer Hospital of Shantou University Medical College, Shantou, 515041 Guangdong China; 2 Department of Radiation Oncology, Cancer Hospital of Shantou University Medical College, Shantou, 515041 Guangdong China; 3 Engineering College, Shantou University, Shantou, 515041 Guangdong China

**Keywords:** Immune checkpoint blockade, Metastatic melanoma, Ligand-receptor pairs, Biomarker

## Abstract

Immune checkpoint blockade (ICB) therapy has transformed the treatment landscape for metastatic melanoma, yet predicting therapeutic response remains a significant challenge. This study hypothesizes that coordinated ligand-receptor (LR) interactions within the tumor microenvironment (TME) critically influence ICB efficacy and proposes that a novel LR pair-based signature score (LRPS) derived from on-treatment samples can predict clinical outcomes. In this study, we analyzed publicly available transcriptomic and clinical datasets comprising 144 patients and 168 on-treatment tumor samples from five independent cohorts (GEO: GSE120575, GSE115821, GSE168204; BioProject: PRJEB23709; dbGaP: phs001919.v1.p1; EGA: EGAD00001005738). We identified seven LR pairs (FLT3-FLT3LG, LY9-LY9, CD5-CD5, CD40LG-ITGA2B/ITGB3, APP-CD74, TNFRSF17-TNFSF13, FCER2-ITGAV/ITGB3) significantly associated with treatment outcomes. LRPS demonstrated strong predictive power, achieving an area under the ROC curve (AUC) exceeding 0.80 in four independent cohorts. Patients in the high-LRPS group exhibited higher ICB response rates (up to 76.2%) and significantly better progression-free survival (PFS) and overall survival (OS) compared with the low-LRPS group. In conclusion, we identified and verified an LRPS signature that provides a theoretical basis for applying such signatures derived from on-treatment tumor samples to predict therapeutic responses to ICB therapies.

**Supplementary Information:**

The online version contains supplementary material available at 10.1007/s12672-026-04396-4.

## Introduction

Although immune checkpoint blockade (ICB) has revolutionized the clinical management of advanced malignancies, including melanoma, durable therapeutic benefits are still confined to a subset of patients [1–3]. Developing reliable predictive biomarkers to identify responders could help minimize adverse effects, prevent acquired resistance, and improve the cost-effectiveness of immunotherapy [4]. These considerations highlight the urgent need for robust molecular signatures to guide patient selection for ICB treatment.

Current predictive frameworks for ICB response in metastatic melanoma incorporate a wide range of biomarkers, including—but not limited to—tumor mutational burden (TMB) [5], NLRP3 inflammasome signatures [6], immune infiltrate composition [7, 8], inflammatory signatures [9], tumor-associated endothelial gene signatures [10], platelet activation cascades [11], and immune checkpoint interaction scores (IMPRES) [12]. Notably, most of these biomarkers are derived from pre-treatment biopsies, which may fail to capture the dynamic microenvironmental changes induced by ICB therapy itself.

Ligand–receptor (LR) interactions within the tumor microenvironment (TME) mediate essential cell-to-cell communication processes that drive oncogenesis. These interactions not only regulate tumor cell proliferation and survival but also orchestrate metastatic progression [13]. Tumor cells communicate with their surrounding microenvironment through direct cell–cell contact and by secreting soluble mediators such as growth factors, cytokines, and chemokines [14]. Moreover, stromal components—including mesenchymal stem cells, cancer-associated fibroblasts, and tumor-associated macrophages—actively modulate malignant phenotypes via bidirectional signaling [14]. For instance, dysregulation of the CXCL16–CXCR6 axis has been shown to promote immunosuppressive macrophage polarization [15]. Nevertheless, whether dynamic LR pair signatures derived from on-treatment tumor samples can predict ICB response outcomes in metastatic melanoma remains largely unexplored.

Although several immune-related signatures have been developed using pre-treatment tumor samples, these approaches may overlook therapy-induced transcriptional reprogramming within the tumor microenvironment. Furthermore, the contribution of ligand-receptor communication dynamics during ICB therapy remains largely unexplored. To address this gap, our study focuses on on-treatment tumor specimens to capture dynamic immune-tumor interactions, aiming to construct a ligand-receptor pair-based signature (LRPS) that more accurately predicts ICB response and patient outcomes. In this study, through analysis of transcriptomic and clinical data from ICB-treated metastatic melanoma patients, we developed an LRPS using ElasticNet penalized Logistic Regression (ENLR) to predict treatment response in on-treatment specimens. This approach not only provides a clinically actionable biomarker but also identifies targetable communication nodes for rational combination immunotherapy design.

## Methods

### Data collection

**Fig. 1:**
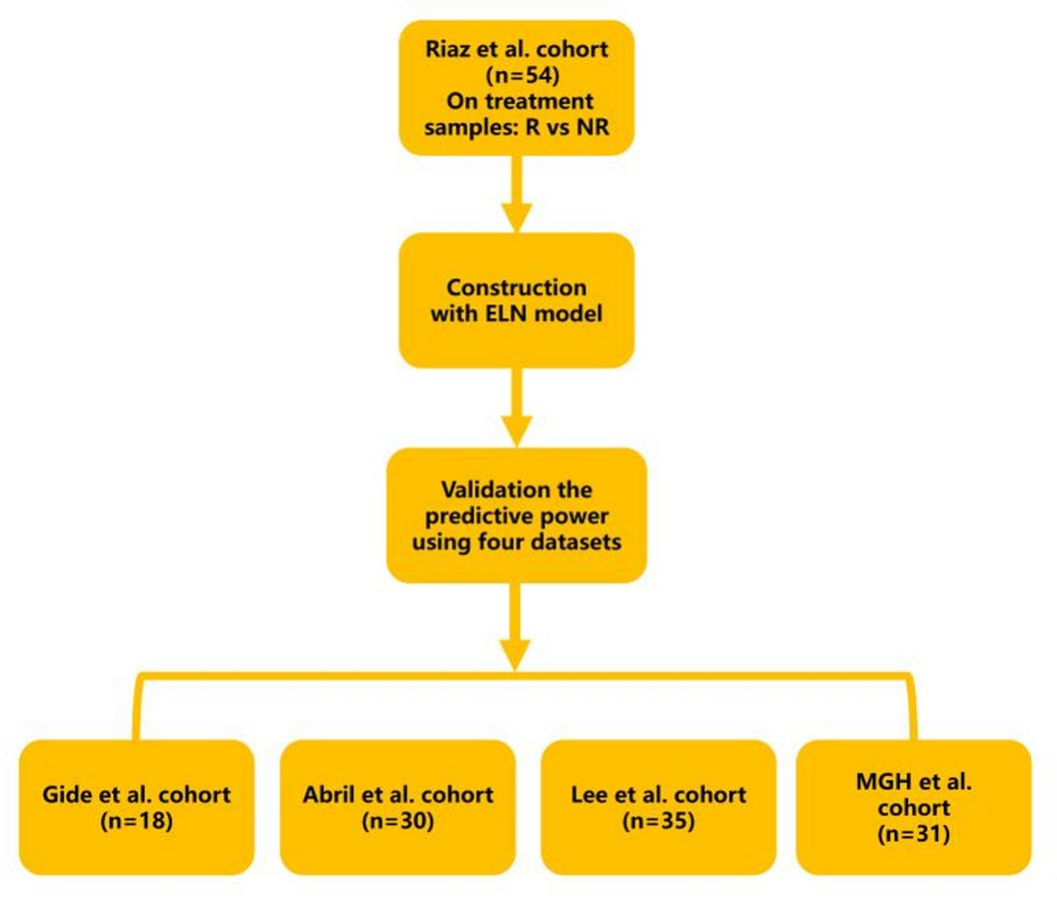
The flow chart of LRPS signature score construction

The workflow of this study is illustrated in Fig. 1. We compiled transcriptomic data from five independent publicly available cohorts of on-treatment tumor biopsies collected from patients with metastatic melanoma, a solid tumor type. These datasets include the Riaz et al. cohort (GEO: GSE120575) [16], the Gide et al. cohort (BioProject: PRJEB23709) [17], the Abril et al. cohort (dbGaP: phs001919.v1.p1) [18], the Lee et al. cohort (EGA: EGAD00001005738) [19], and the MGH et al. cohort (GEO: GSE115821 and GSE168204). We compiled transcriptomic data from five independent cohorts of on-treatment metastatic melanoma samples, defined as biopsies collected after initiation of ICB therapy (anti-PD-1/PD-L1 ± anti-CTLA-4). Samples were excluded from analysis based on the following criteria: (1) absence of RNA-seq data; (2) missing clinical response information; or (3) duplicated tumor specimens from the same timepoint in individual patients. Summary of the five melanoma cohorts and their on-treatment sampling time points is provided in Supplementary Table S1.

Gene expression was quantified using transcripts per million (TPM) for each gene. TPM normalization was applied because it accounts for both sequencing depth and gene length, allowing for more comparable expression levels across datasets and cohorts. According to RECIST criteria [20], responders (R) were defined as individuals who achieved complete response (CR), partial response (PR), or progression-free survival (PFS) of over 180 days. Non-responders (NR) were classified as those with PFS under 180 days accompanied by progressive disease (PD).

### Ligand-receptor pair selection

LR pairs were curated from published literature [21, 22]. The selection of these pairs was grounded in the biological relevance of these interactions in the immune response. The comprehensive list of selected LR pairs is presented in Supplementary Table S2.

### Calculation of signature scores

In this study, we utilized the Riaz et al. cohort as the training dataset, while the remaining cohorts served as validation datasets. We computed the score for each LR pair in on-treatment metastatic melanoma samples from the Riaz et al. cohort using the single-sample gene set enrichment analysis (ssGSEA) algorithm. We performed Elastic Net regression using the R software with the glmnet package, applying an Elastic-Net penalized logistic regression model to identify the LR pairs most strongly associated with response to ICB therapy. To further refine our analysis, we computed a weighted average for each sample, using the effect size as a weighting factor for the LR pairs most associated with ICB therapy response. This weighted average was defined as the LR pair-based signature score (LRPS).

To minimize the risk of overfitting, five-fold cross-validation was performed on the training set [23]. To correct for class imbalance in our dataset, we incorporated a cost-sensitive learning approach during model training, which was implemented via the cv.glmnet, a function from the glmnet package. Specifically, we applied a prior probability-based offset in the binomial logistic regression model. This offset was computed as a log-odds correction term using the empirical class frequencies and a predefined classification threshold (τ = 2/3). The model was trained using cross-validation (nfolds = 5) to optimize the regularization parameter (lambda) with respect to the area under the curve (AUC). By incorporating the correction term, the model was encouraged to assign greater importance to the minority class without modifying the training data or employing resampling techniques. This strategy enabled a more balanced and robust model performance under imbalanced class conditions.

The predictive performance of the LRPS model was evaluated by generating receiver operating characteristic (ROC) curves using the 'pROC' package in R, with the AUC used to quantify its discriminatory accuracy. Using data from the Riaz et al. cohort, the optimal threshold value was determined using Youden’s index during ROC analysis. Finally, the odds ratios for individual samples were calculated based on their LRPS.

### Statistical analysis

All statistical analyses and graphical visualizations were conducted using R software (version 4.2.0) and GraphPad Prism (version 8.0). The one-tailed Wilcoxon rank-sum test was applied to assess statistical differences between the R and NR groups. We calculated the sample’s odds ratio (OR) based on the signature score. Samples were classified into high and low group use mean value of samples’ OR as cutoff. Kaplan–Meier (KM) survival analysis, along with the log-rank test, was used to evaluate differences in survival outcomes between the high and low OR groups. To further assess the impact of the signature score on survival, Cox proportional hazards regression models were applied to estimate the hazard ratio (HR) and corresponding 95% confidence intervals (CI) within each cohort. The proportional hazards assumption was tested using the Global Schoenfeld residuals test. Given the exploratory nature of this biomarker study, we used a significance threshold of 0.05 when reporting *p*-values, with trends between 0.05 and 0.1 reported to avoid arbitrary dichotomization of significance [24].

## Results

### Patient cohorts

In the Riaz et al. dataset, a total of 54 biopsies at the on-treatment timepoint were included in the analysis. Of these, 21 biopsies were from R, and 33 were from NR. All patients received anti-PD-1 monotherapy. In the Gide et al. dataset, 17 patients with 18 on-treatment biopsies (11 R and 7 NR) received anti-PD-1 monotherapy. The median age of the patients was 56 years, with 41% over 60 years old. Additionally, 53% of the patients were male. In the Lee et al. dataset, 23 patients with 35 on-treatment biopsies (6 R and 29 NR) were treated with anti-PD-1 monotherapy, including nivolumab or pembrolizumab. The Abril-Rodriguez cohort included 30 on-treatment tumor samples (13 R and 17 NR). All patients received PD-L1 monotherapy (pembrolizumab). The MGH cohort included 31 on-treatment samples (5 R and 26 NR). All patients received anti-PD-1/PD-L1 monotherapy. The baseline characteristics of the patients are presented in Supplementary Table S3.

**Fig. 2:**
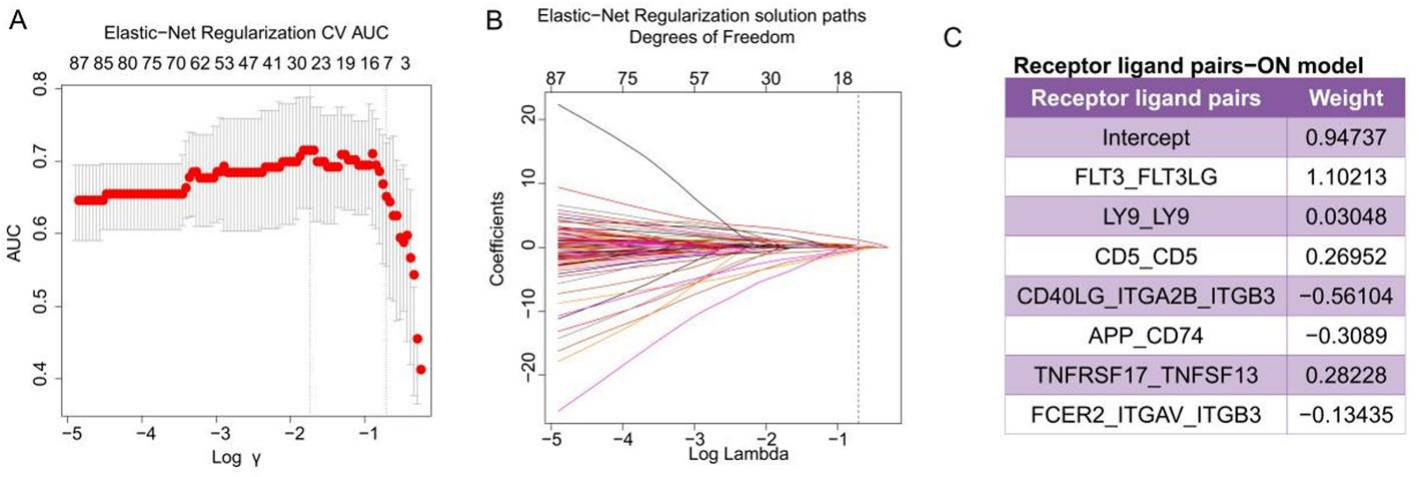
LRPS signature for on-treatment samples from the Riaz et al. cohort. **A**, **B**. The modelʹs training parameter selection process was used to generate the Riaz et al. on-treatment samples to generate the LRPS signature. To avoid overfitting, 5‐fold cross‐validation was performed with the parameter setting as ʺtype.measure = auc, family = ‘binomial’.ʺ **C**. LRPS signatures consisted of seven selected frames associated with the effect sizes (variable weights) from the elasticnet penalized logistic regression model

As illustrated in Fig. 1, the Riaz et al. cohort, comprising 54 on-treatment metastatic melanoma samples, was utilized as the training set, while other cohorts served as validation sets. The ssGSEA algorithm was employed to compute scores for LR pairs within the Riaz et al. training dataset. An elastic net algorithm was then applied to identify LR pairs significantly associated with response to ICB therapy. To mitigate overfitting, we implemented five-fold cross-validation and used a cost-sensitive algorithm (Fig. 2A, B). Ultimately, as shown in Fig. 2C, seven LR pairs associated with ICB therapy response were identified: FLT3-FLT3LG, LY9-LY9, CD5-CD5, CD40LG-ITGA2B/ITGB3, APP-CD74, TNFRSF17-TNFSF13, and FCER2-ITGAV/ITGB3. Effect size was subsequently used as a weighting factor to calculate the weighted average of the ssGSEA scores for these seven LR pairs, defining the LRPS for each sample.

### LRPS is an effective predictor of response and prognosis in ICB therapy for on-treatment samples

**Fig. 3:**
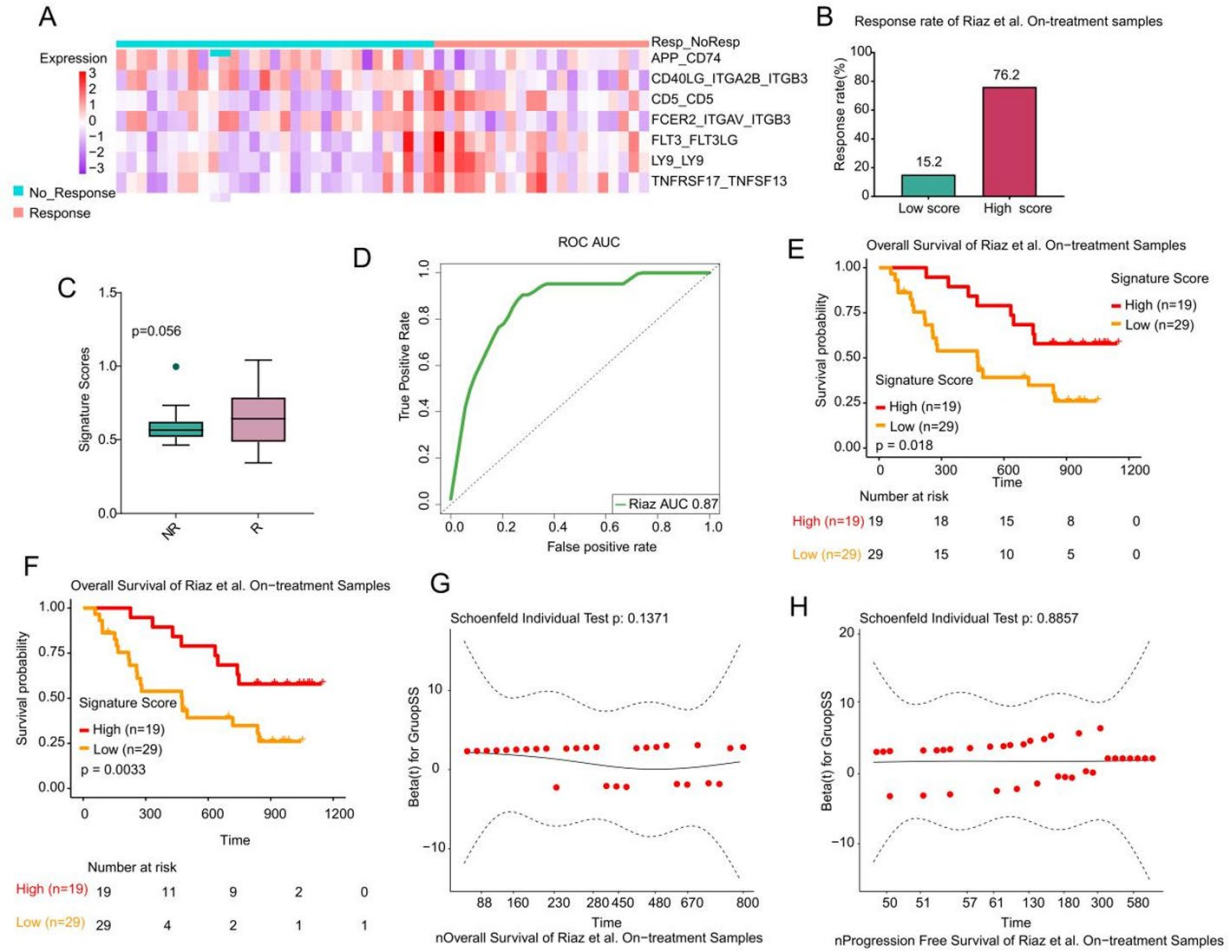
LRPS signature for on-treatment samples from Riaz et al. cohort. **A**. Heatmap representing the single sample gene set enrichment analysis value of on-treatment nonresponders (NR) and responders (R) in the Riaz et al. cohort. Nonresponders are presented with the number of samples on the left side, and responders are presented with the number of samples on the right side. **B**. The response rate to immunotherapy in low- and high-LRPS scores groups in the Riaz et al. cohort. **C**. Comparison of LRPS scores between high- and low-LRPS groups in on-treatment samples from the Riaz et al. cohort. **D**. Receiver operating correlation curve and area under the curve of LRPS signatures for on-treatment samples from the Riaz et al. cohort. **E**, **F** Kaplan–Meier curves of PFS and OS for pretreatment samples based on LRPS signature scores for the Riaz et al. cohort. The two-sided log-rank test compared high and low subgroups based on the mean of on-treatment samples odds ratio (OR) as cutoff. Hazard ratio (HR) was calculated and shown with confidence interval (CI). **G**, **H** Graphical assessment of the proportional hazard assumption of the LRPS signature in on-treatment samples

As illustrated in Fig. 3A, the heatmap presents the distribution of ssGSEA scores for seven LR pairs within the Riaz et al. cohort. The high-LRPS group demonstrated a response rate of 76.2% to ICB therapy, which markedly surpassed the 15.2% observed in the low-LRPS group (Fig. 3B). Although not statistically significant (*p* = 0.056), the LRPS in the R group was higher than in the NR group, suggesting a trend toward greater response with elevated LRPS values (Fig. 3C). ROC analysis further highlighted LRPS’s predictive power, with an AUC of 0.87 in forecasting response to ICB therapy in metastatic melanoma within the training set (Fig. 3D). Moreover, survival analyses demonstrated that patients with high LRPS experienced extended overall survival (OS) and PFS (*p* < 0.05), underscoring LRPS’s prognostic value (Fig. 3E, F). Additionally, we performed the Global Schoenfeld test to evaluate whether the score acted as a time-dependent covariate. The tests showed *p*-values greater than 0.05 (Fig. 3G and H), suggesting that the assumption of proportional hazards was not violated. The LRPS score did not vary over time when predicting OS and PFS, indicating it is not a time-dependent covariate in Riaz et al. cohort.

### Validation of LRPS prediction performance

**Fig. 4:**
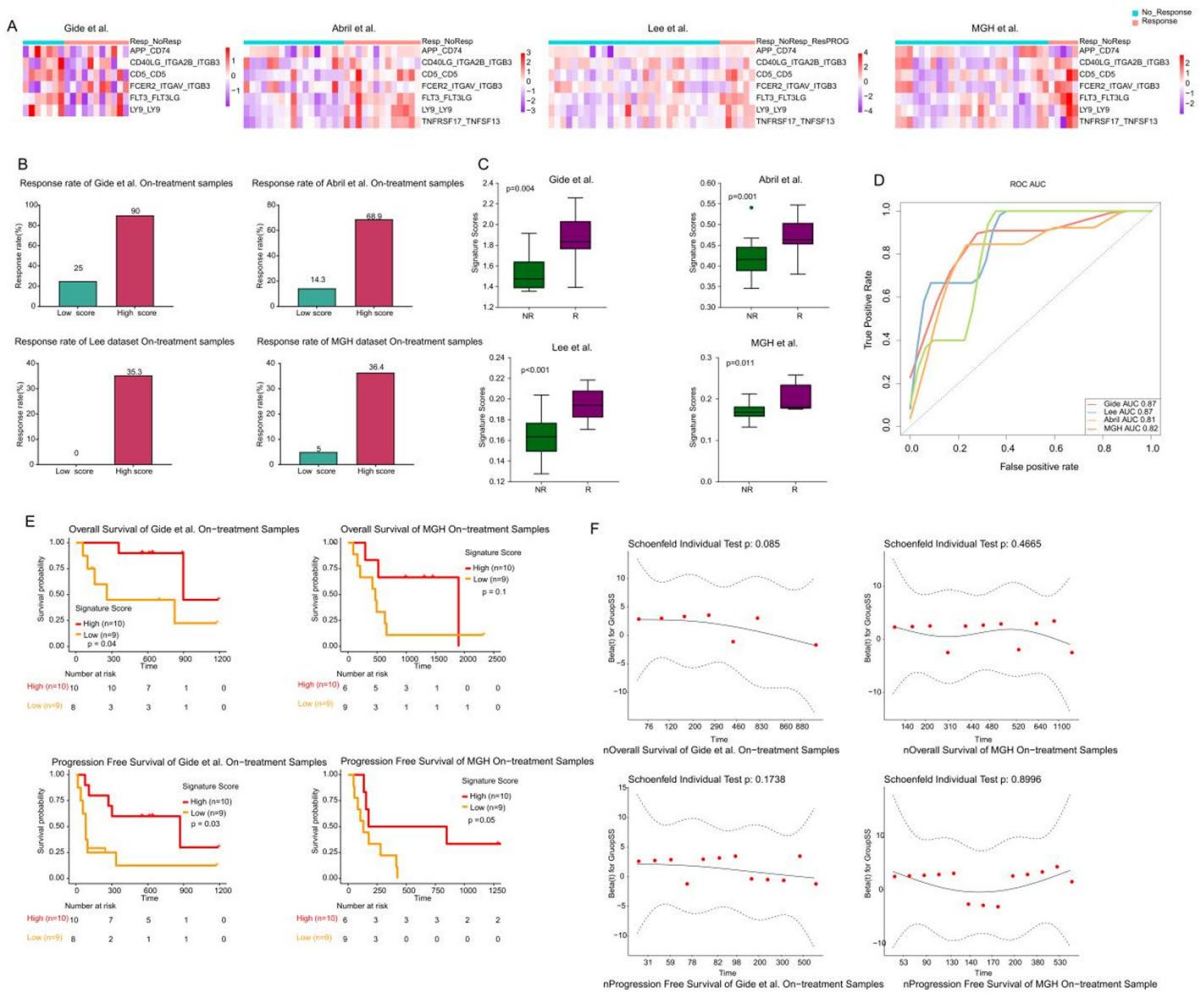
LRPS signature for on-treatment samples from validation cohorts. **A**. Heatmap representing the single sample gene set enrichment analysis value of on-treatment nonresponders (NR) and responders (R) in the Gide et al., Abril-Rodriguez et al., Lee et al. cohorts, and MGH cohorts. **B**. The response rate to immunotherapy in low- and high-LRPS scores groups in the Gide et al., Abril-Rodriguez et al., Lee et al. and MGH cohorts. **C**. Comparison of LRPS scores between high- and low-LRPS groups in on-treatment samples from the Gide et al., Abril-Rodriguez et al., Lee et al. and MGH cohorts. **D**. Receiver operating characteristic (ROC) curve and area under the curve of LRPS signatures for on-treatment samples from the Gide et al., Abril-Rodriguez et al., Lee et al. and MGH cohorts. **E, F**. Kaplan–Meier curves of PFS or OS for on-treatment samples based on LRPS signature scores for Gide et al. and MGH cohort. The two-sided log-rank test compared high and low subgroups based on the mean of on-treatment samples odds ratio (OR) as cutoff. Hazard ratio (HR) was calculated and shown with confidence interval (CI). **G, H** Graphical assessment of the proportional hazard assumption of the LRPS signature in on-treatment samples

We utilized heatmaps to illustrate the distribution of ssGSEA scores for LR pairs within the R and NR groups across validation cohorts (Fig. 4A). In each cohort, we observed a significantly higher response rate to ICB therapy in the high-LRPS group compared to the low-LRPS group (Fig. 4B). In all four validation cohorts, LRPS scores were significantly higher in the R group than in the NR group (*p* < 0.05) (Fig. 4C). ROC analysis indicated that LRPS was a robust predictor of treatment response, achieving AUC values of 0.8 or higher across the cohorts (Fig. 4D). Additionally, in the Gide et al. and MGH cohorts, patients with high LRPS experienced longer OS and PFS compared to the low-LRPS group (Fig. 4E, F). In the Gide cohort, both OS and PFS differences were statistically significant, indicating a strong prognostic relevance of LRPS in this population. Although the OS difference in the MGH cohort did not reach statistical significance, a clear trend toward improved survival was still observed in the high-LRPS group, suggesting consistent biological relevance across cohorts. To evaluate whether LRPS functioned as a time-dependent covariate, we performed the Global Schoenfeld test in both cohorts. All tests returned *p*-values greater than 0.05 (Fig. 4G, H), indicating that the proportional hazards assumption was upheld. This suggests that the predictive ability of the LRPS remained stable over time and does not depend on time-varying effects. Together, these findings reinforce the robustness of LRPS as a time-independent prognostic marker for OS and PFS across independent cohorts, further supporting its potential utility in clinical stratification for immune checkpoint blockade therapy.

**Fig. 5:**
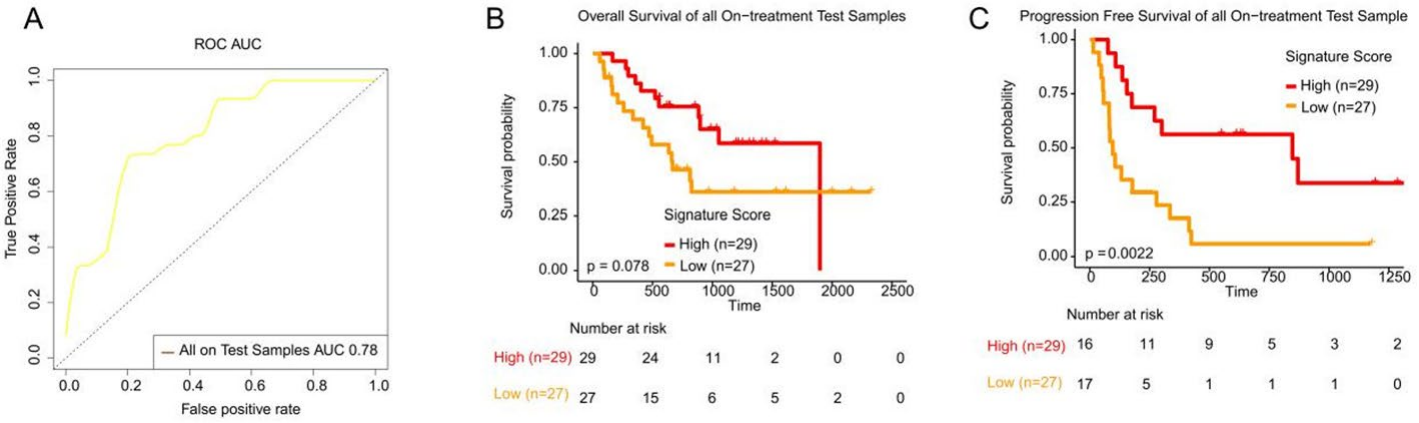
LRPS signature for all on-treatment samples from test cohorts. **A**. Receiver operating characteristic (ROC) curve and area under the curve of LRPS signatures for all on-treatment samples from test cohorts. **B**. Kaplan–Meier curves of PFS or OS for on-treatment samples based on LRPS signature scores for all on-treatment samples from test cohorts. The two-sided log-rank test compared high and low subgroups based on the mean of on-treatment samples odds ratio (OR) as cutoff. Hazard ratio (HR) was calculated and shown with confidence interval (CI)

We further combined the four validation cohorts, examining LRPS’s ability to predict ICB therapy response across test cohorts. ROC analysis revealed an AUC of 0.78, confirming the predictive strength of LRPS across all test cohorts (Fig. 5A). Although high LRPS was associated with extended OS in all cohorts, this difference did not reach statistical significance (*p* = 0.078) (Fig. 5B). However, high LRPS was significantly associated with improved PFS (*p* < 0.05) (Fig. 5C).

We performed a chi-square test to assess the association between the LRPS signature and clinical variables, including tumor grade, tumor type (cutaneous vs. non-cutaneous melanoma), mutation type, age, and sex. No statistically significant correlations were found between these variables and the immune-metabolic signature (Supplementary Table S5), indicating that the LRPS signature was not significantly influenced by these clinical factors. These findings are limited by the available clinical data, which did not include treatment context, ICB doses, time intervals between diagnosis and sample collection, or disease stage. These factors may potentially affect the signature, but their absence from the dataset prevents further analysis of their impact.

### Impact of responder proportion on LRPS model performance

Although LRPS demonstrated robust predictive performance across validation cohorts, we further examined the impact of responder proportion on model performance metrics to evaluate its clinical applicability. To validate the generalizability of the LRPS model, its performance was evaluated across five independent cohorts, including one training and four test cohorts. As summarized in Supplementary Table S4, the model achieved robust predictive performance in the training cohort (Riaz et al.) with an AUC of 0.869 and an F1 score of 0.792. In the validation cohorts, the model maintained high AUC values ranging from 0.814 to 0.874. Notably, the best performance was observed in the Gide et al. cohort (AUC = 0.870, F1 = 0.909), which had a relatively balanced responder-to-non-responder ratio (11:7, 61%). In contrast, the Lee and MGH cohorts, both of which exhibited strong class imbalance with responders comprising only 16–17% of samples, showed lower precision (0.375 and 0.385, respectively) and F1 scores (0.545 and 0.556), despite achieving perfect recall (1.000). This suggests that the model successfully identified most responders but at the cost of increased false positives in imbalanced datasets. The Abril-Rodriguez cohort demonstrated consistent performance with an AUC of 0.814, precision of 0.786, and F1 score of 0.815. Collectively, these results indicate that while the LRPS model is generally robust across multiple cohorts, its precision could be affected by responder prevalence, highlighting the importance of class distribution in model evaluation and deployment.

### LRPS is an independent prognostic factor for metastatic melanoma

**Fig. 6:**
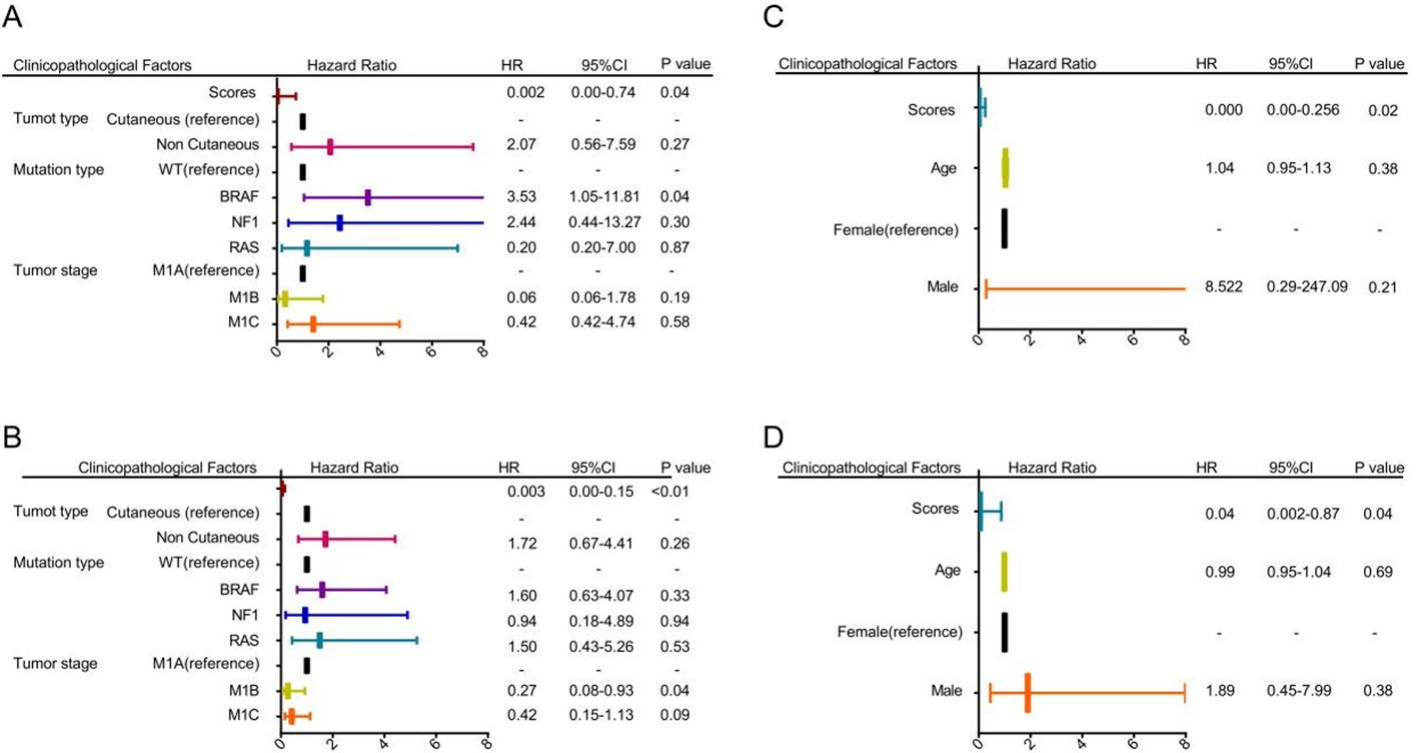
Results of Cox proportional hazards regression for PFS and OS analysis using LRPS signature scores for on-treatment samples. If the p-value is less than 0.05, it is shown in bold font

We conducted a multivariate Cox regression analysis in the training dataset from the Riaz et al. cohort, including four variables: LRPS, tumor type, mutation type, and tumor stage, to determine whether LRPS is an independent factor for predicting the prognosis of patients with metastatic melanoma. Our results indicate that LRPS is an independent prognostic marker for OS (HR = 0.002, *p* = 0.04) and PFS (HR = 0.003, *p* < 0.01, Fig. 6A, B). Furthermore, we validated the role of LRPS as an independent prognostic factor for predicting OS and PFS in patients with metastatic melanoma in Gide et al. cohort (Fig. 6C, D).

## Discussion

Since the approval of ipilimumab, the first checkpoint inhibitor targeting CTLA-4, immunotherapy, particularly with anti-PD-1 and anti-PD-L1 antibodies, has demonstrated remarkable clinical efficacy in treating various cancers and has become a cornerstone of ICB therapy [25]. However, a notable proportion of patients still fail to achieve satisfactory responses to these treatments [26]. To address this challenge, we developed a scoring system based on seven LR pairs that calculates the LRPS from on-treatment samples, which may provide insights for predicting responses to ICB therapy in metastatic melanoma. Our findings suggest that patients with high LRPS values exhibit improved treatment response rates across multiple cohorts. ROC analysis revealed that LRPS achieved AUC values exceeding 0.8 in predicting ICB therapy responses in specific cohorts. Furthermore, elevated LRPS was associated with improved prognosis, highlighting its dual role as a predictive and prognostic biomarker. To place our findings in the context of previously published predictive models, we compared the performance of LRPS with several established biomarkers and transcriptomic signatures for ICB response prediction in metastatic melanoma, such as inflammatory or endothelial gene signatures [9, 10]. Reported AUC values for these models typically range between 0.60 and 0.80 across independent validation cohorts. In contrast, the LRPS model achieved AUCs exceeding 0.80 in four independent on-treatment datasets, indicating comparable or superior predictive accuracy. Unlike models derived from pre-treatment biopsies, LRPS is based on on-treatment samples, capturing the dynamic immune–tumor interactions induced by ICB therapy. This distinction likely contributes to its enhanced prognostic performance. Moreover, LRPS integrates ligand–receptor communication networks, offering mechanistic insights into immune modulation that complement existing immune infiltration– or pathway-based models. Collectively, these comparisons highlight the added value of LRPS in predicting ICB efficacy and underscore its potential for guiding personalized immunotherapy.

Our study underscores the value of RNA sequencing when on-treatment tumor samples are available. The LRPS may serve as a practical tool to stratify patients according to their likelihood of benefiting from ICB therapy. Specifically, patients with higher LRPS scores are more likely to achieve favorable therapeutic outcomes, whereas those with lower LRPS scores may require combination strategies (e.g., chemotherapy or radiotherapy) to remodel the immunosuppressive tumor microenvironment and enhance immune activation. For this subgroup, alternative therapeutic approaches should also be explored.

Among the seven LR pairs identified, FLT3-FLT3LG had the highest weight in the elastic net model. FLT3 and its ligand FLT3LG are critical for dendritic cell (DC) development and activation [27], processes essential for antigen presentation and adaptive immune response initiation. FLT3LG promotes DC differentiation and expansion, potentially enhancing T cell priming and cytotoxic T lymphocyte (CTL) activation against tumors [28]. This mechanism may improve immune recognition of tumor cells and synergize with ICB therapy.

Lymphocyte Antigen 9 (LY9/CD229), highly expressed on NKT cells [29]. Regulates NKT cell differentiation. LY9 deletion promotes NKT cell differentiation and expands innate CD8⁺ T cell populations [30]. Given the opposing roles of type I (pro-inflammatory) and type II (immunosuppressive) NKT cells in tumor immunity, LY9 upregulation may skew the balance toward type I NKT cells, thereby augmenting anti-tumor responses [29].

CD5⁺ dendritic cells (DCs) are critical for T cell priming and antitumor immunity. Recent studies demonstrate that CD5⁺ DCs correlate with improved survival in melanoma patients and expand during ICB therapy [31]. Loss of CD5 impairs T cell-mediated tumor clearance, underscoring its essential role in ICB efficacy.

Elevated CD40LG expression in tumors is associated with M2 macrophage infiltration and immunosuppression [32–34]. Concurrently, the integrin heterodimer ITGA2B/ITGB3 (αIIbβ3) may promote tumor cell adhesion to extracellular matrix (ECM) components, creating physical barriers to immune infiltration [35, 36]. Together, this axis may foster an immune-evasive microenvironment, reducing ICB efficacy.

Amyloid precursor protein (APP) overexpression activates MAPK signaling to drive tumor invasion and metastasis [37]. Tumor-derived APP binding to CD74 may shift the tumor microenvironment toward immunosuppression [38], suggesting that targeting the APP-CD74 axis could restore immune activity and improve ICB outcomes.

Elevated TNFRSF17 (BCMA) and TNFSF13 (APRIL) expression in high-LRPS patients aligns with evidence linking plasma cell infiltration to improved immunotherapy outcomes [8]. TNFSF13 binding to BCMA and TACI promotes B cell activation, plasma cell differentiation, and long-term survival [39], facilitating antibody-dependent cellular phagocytosis (ADCP) of tumor cells [39, 40]. This mechanism may synergize with ICB to amplify antitumor immunity.

FCER2 (CD23) suppresses plasma cell differentiation and antibody production by inhibiting class-switched B cells [41]. Meanwhile, ITGAV/ITGB3 (αVβ3 integrin) drives TGF-β-mediated stromal remodeling and PD-L1 upregulation [35, 36]. Their synergy may establish a feedforward loop enhancing immune evasion. While individual LR pairs included in the LRPS showed variable levels of prognostic value, the integrated LRPS model, consisting of all seven pairs, demonstrated significantly improved predictive performance. This highlights the synergistic effect among LR pairs and suggests that the complete signature provides a more reliable prognostic and potentially therapeutic tool than any single pair or subset alone.

Our study has several limitations that should be acknowledged. First, the retrospective design, which relies on aggregated transcriptomic data from publicly available datasets, introduces inherent constraints such as potential selection bias and limited patient diversity. Technical variability across sequencing platforms may persist despite normalization efforts, and incomplete annotation of critical clinical covariates (e.g., pretreatment regimens) further limits biological interpretation and generalizability. Second, although the observed trends were consistent, the borderline statistical significance in the training cohort warrants validation in larger, prospective studies. The extremely low hazard ratios for both OS and PFS indicate a strong separation between high- and low-risk groups. While this may reflect excellent discriminative capacity of the selected features, such extreme values should be interpreted with caution, as they may be influenced by data distribution, sample size, and high-dimensional modeling effects. Third, the predictive precision of the LRPS model decreased in cohorts with severe class imbalance, where perfect recall was accompanied by increased false-positive rates, emphasizing the need to account for responder prevalence in clinical implementation. Finally, mechanistic understanding of specific LR pairs remains incomplete and requires further experimental validation. While this study focused on metastatic melanoma, future investigations will aim to evaluate the LRPS signature in other ICB-treated malignancies such as renal cell carcinoma and non-small cell lung cancer to assess its cross-cancer applicability.

In conclusion, the LRPS derived from on-treatment tumor samples holds promise as a predictive biomarker for ICB therapy response in metastatic melanoma. Patients with high LRPS consistently demonstrate improved therapeutic outcomes and prognosis across multiple cohorts, supporting its potential clinical utility. Collectively, this study provides a foundation for incorporating LRPS into precision immunotherapy strategies, although further prospective validation is required.

## Supplementary Information

Below is the link to the electronic supplementary material.

Supplementary Material 1.

## Data Availability

The datasets analyzed in this study are currently available in the respective online repositories. The specific access links or accession numbers are as follows: Riaz et al. dataset, available in the GEO database (https://www.ncbi.nlm.nih.gov/geo/), accession number GSE120575. Gide et al. dataset, available in the BioProject database (https://www.ncbi.nlm.nih.gov/bioproject), accession number PRJEB23709. Abril et al. dataset, available in the dbGaP database (https://www.ncbi.nlm.nih.gov/gap/), accession number phs001919.v1.p1. Lee et al. dataset, available in the EGA database (https://ega-archive.org/?lang=zh), accession number EGAD00001005738. MGH et al. dataset, available in the GEO database (https://www.ncbi.nlm.nih.gov/geo/), accession numbers GSE115821 and GSE168204.

## Abbreviations

ICB: Immune checkpoint blockade
IMPRES: Immune checkpoint interaction scores
LR: Ligand-receptor
ENLR: ElasticNet penalized Logistic Regression
TPM: Transcripts per million
R: Responders
CR: Complete response
PR: Partial response
PFS: Progression-free survival
NR: Non-responders
PD: Progressive disease
ssGSEA: Single-sample gene set enrichment analysis
LRPS: LR pair-based signature score
AUC: Area under the curve
ROC: Receiver operating characteristic
KM: Kaplan–Meier
CI: Confidence intervals
OS: Overall survival
DC: Dendritic cell
CTL: Cytotoxic T lymphocyte
ECM: Extracellular matrix
APP: Amyloid precursor protein
ADCP: Antibody-dependent cellular phagocytosis
